# SnSe_2_ Field-Effect Transistor with High On/Off Ratio and Polarity-Switchable Photoconductivity

**DOI:** 10.1186/s11671-019-2850-0

**Published:** 2019-01-09

**Authors:** Hong Xu, Jie Xing, Yuan Huang, Chen Ge, Jinghao Lu, Xu Han, Jianyu Du, Huiying Hao, Jingjing Dong, Hao Liu

**Affiliations:** 1 School of Sciences, China University of Geosciences, Beijing, 100083 China; 2 Beijing National Laboratory for Condensed Matter Physics, Institute of Physics, Chinese Academy of Sciences, Beijing, 100190 China

**Keywords:** Field-effect transistor, SnSe_2_, Photoconductivity, On/off ratio

## Abstract

SnSe_2_ field-effect transistor was fabricated based on exfoliated few-layered SnSe_2_ flake, and its electrical and photoelectric properties have been investigated in detail. With the help of a drop of de-ionized (DI) water, the SnSe_2_ FET can achieve an on/off ratio as high as ~ 10^4^ within 1 V bias, which is ever extremely difficult for SnSe_2_ due to its ultrahigh carrier density (10^18^/cm^3^). Moreover, the subthreshold swing and mobility are both improved to ∼ 62 mV/decade and ~ 127 cm^2^ V^−1^ s^−1^ at 300 K, which results from the efficient screening by the liquid dielectric gate. Interestingly, the SnSe_2_ FET exhibits a gate bias-dependent photoconductivity, in which a competition between the carrier concentration and the mobility under illumination plays a key role in determining the polarity of photoconductivity.

**Electronic supplementary material:**

The online version of this article (10.1186/s11671-019-2850-0) contains supplementary material, which is available to authorized users.

## Introduction

Due to the quantum confinement effect, two-dimensional (2D) atomically layered materials (ALMs) behave very differently from their 3D bulk counterparts, exhibiting some unique and fascinating electronic, optical, chemical, magnetic, and thermal properties [1]. 2D ALMs provide an attracting platform for fundamental physical and chemical research at the limit of a single atom or few-layer thickness. Moreover, ALMs could be flexibly integrated with other devices, offering a bigger room or freedom to develop novel functions beyond the reach of the existing materials. Over the past decade, the 2D ALMs have been widely investigated and found potential applications in fields such as sensors, energy, and environment [2, 3].

Recently, as an important member of the IV-VI group, tin diselenide (SnSe_2_) has drawn much attention. SnSe_2_ has a hexagonal CdI_2_-type crystal structure, in which the Sn atoms are sandwiched by two layers of hexagonally packed Se atoms with space group $\mathrm{p}\overline{3}\mathrm{m}1$ [4]. Unlike transitional metal dichalcogenides (TMDs), SnSe_2_ possesses a narrower bandgap with indirect band gap characteristic within the entire thickness range from the bulk to the monolayer, resulting from outer p electrons of Sn involved in the structural bonding unlike d electrons of Mo or W in MoS_2_ or WS_2_ [5]. SnSe_2_ has been investigated to have excellent properties in thermoelectrics, phase change memory, lithium-ion batteries, and various electronic logic devices [4, 6–9]. Especially, SnSe_2_ has a higher electron affinity (5.2 eV) and therefore has a special application in fabricating tunneling field-effect transistors (FETs) [9–11]. Pan et al. systematically investigated FETs based on mechanically exfoliated SnS_2 − *x*_Se_*x*_ crystals with varying selenium content [12]. They found that the drain-source current (*I*_d_) cannot be completely turned off with the Se content reaching *x* = 1.2 or above. Later Su et al. have fabricated a SnSe_2_ MOSFET with high drive current (160 μA/μm) at 300 K with the same result of no “OFF” state [13]. The main reason for the difficulty in obtaining “OFF” state of SnSe_2_ FET device is the ultrahigh electron density (10^18^ cm^−3^ in bulk SnSe_2_, compared with 10^16^ cm^−3^ in MoS_2_) [14, 15]. Therefore, effective modulation of transport of carriers in SnSe_2_ FETs is a challenging job. Bao et al. successfully turned off *I*_d_ and obtained an on/off ratio of 10^4^ at room temperature when using HfO_2_ as a back gate combined with a top capping layer of polymer electrolyte. However, the performance of SnSe_2_ cannot survive several sweepings due to the irreversible structural transition caused by Li^+^ intercalation into the interlayer of SnSe_2_ [16]. Guo et al. achieved a higher current on/off ratio of 10^5^ with a threshold voltage of − 100 V by thinning the SnSe_2_ flake to 6.6 nm [17]. However, the work temperature is only 78 K, which is not convenient for practical application. An alternative way to enhance the modulation of the transport of carriers in FETs is to deposit a high-k dielectric layer as a top gate, such as HfO_2_ and Al_2_O_3_ [18, 19]. However, the high deposition temperature will change the properties of SnSe_2_ layer and further deteriorate the device performance. Employing a solid polymer electrolyte gate to modulate the carrier density is an attractive method owing to the highly efficient control of the electric double layer (EDL) formed at the interface between the electrolyte and the semiconductor [20–22]. But sluggish ionic migration process requires low-bias sweeping rates to match. So, a simple, efficient, and practical method to modulate the carriers of SnSe_2_ is highly demanding.

In this work, we employed only a drop of de-ionized (DI) water as a solution top gate and successfully switched off the channel current at 300 K. Moreover the on/off ratio could reach ~ 4 orders controlled by a small gate voltage of less than 1 V. More strikingly, the SnSe_2_ device exhibits an interesting bias-dependent negative and positive photoconductivity, in which the possible working mechanism has been analyzed.

## Experiments

The SnSe_2_ flake was obtained from high-quality bulk crystals by mechanical exfoliation. Then, it was transferred onto a Si wafer covered with 100 nm SiO_2_. The detailed exfoliation and transfer method is described in Huang’s paper [23]. After the transfer, optical microscopy was used to identify selected flakes, and the accurate thickness was measured by atomic force microscopy. The SnSe_2_ FETs were fabricated by a standard photolithography. Ti/Au (5/50 nm) contact was deposited by thermal evaporator, followed by in situ annealing at 200 °C in high vacuum (10^−5^ Pa) to improve the metallic contact. For DI water top-gated FETs, an additional polymer layer (polymethyl methacrylate (PMMA) type 950 A5) was deposited on the devices (spin coating at 3000 rpm, thickness ∼400 nm), baked at 180 °C for 2 mins, and patterned by UV photolithography to open windows for contact between the water drop and the device channel.

Electrical characterization was performed by a Keithley sourcemeter 2634B on a four-probe station (Signatone). A laser diode with a wavelength of 532 nm was employed as a light source with a power density of 1 mW/mm^2^ to examine the photoelectric performance of SnSe_2_ FET. The time response was recorded by an oscilloscope MDO3000.

Optical images were obtained using an optical microscope (XTZ-2030JX with a CCD camera). Raman spectrum was performed in the Renishaw in Via Raman Microscope at room temperature with 532-nm laser excitation. AFM characterization was taken by a microscope of Bruker Multimode 8.

## Results and Discussion

**Fig. 1:**
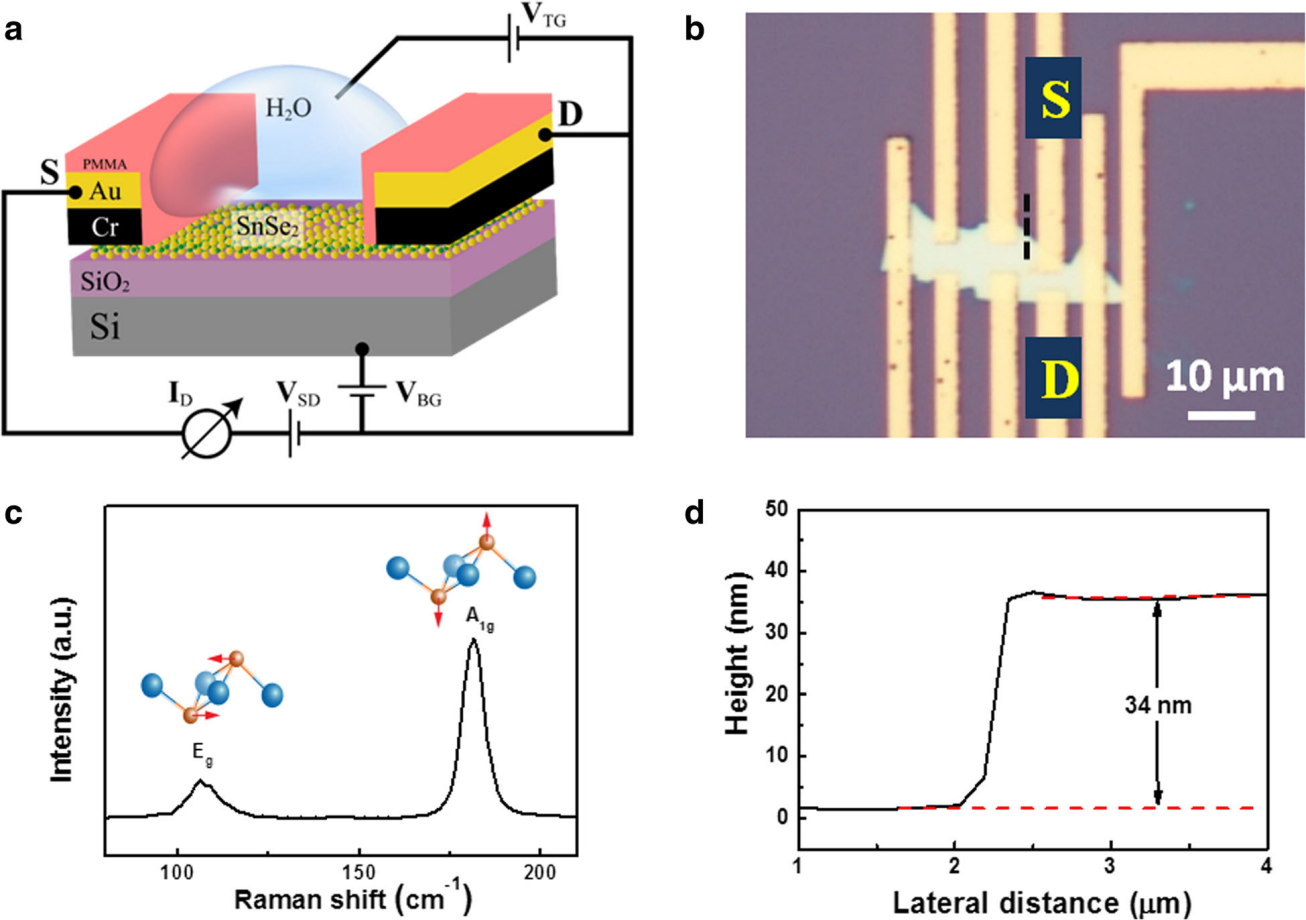
An illustration of SnSe_2_ phototransistor device and some basic characterizations about the SnSe_2_ flake. **a** Schematic illustration of a SnSe_2_ field-effect transistor device. **b** Optical image of a SnSe_2_ flake with S and D denoting the source and drain electrodes under study, respectively. **c** Raman spectrum of a SnSe_2_ flake. **d** A height profile extracted from the black dotted line (shown in Fig. 1**b**) in AFM measurement

Figure 1a shows a schematic diagram of SnSe_2_ FET device. The contacts are covered by a layer of PMMA (type 950 A5) to electrically isolate them from the top gate, which consists of a drop of DI water dripped from a pipette. The device can be gated by a top gate voltage (*V*_tg_) applied to an electrode in contact with the DI water drop or by a back gate voltage (*V*_bg_) applied via the SiO_2_ support. The optical image of SnSe_2_ flakes with patterned electrodes is shown in Fig. 1b. The source-drain gap is about 2 μm. Raman spectroscopy was used to characterize SnSe_2_ material, as shown in Fig. 1c. The fingerprint peaks at 187 cm^−1^ and 112 cm^−1^ corresponds to the out-of-plane *A*_1g_ mode and in-plane *E*_g_ mode, respectively, which agrees well with others’ reports. However, it is difficult to determine the thickness for SnSe_2_ from the position of Raman peak. Unlike MoS_2_, the thickness-dependent characteristic of Raman peak position is not clear [24–26]. So, we adopted atomic force microscopy (AFM) to measure the flake thickness directly. As shown in Fig. 1d, the thickness of SnSe_2_ flake is about 34 nm.

**Fig. 2:**
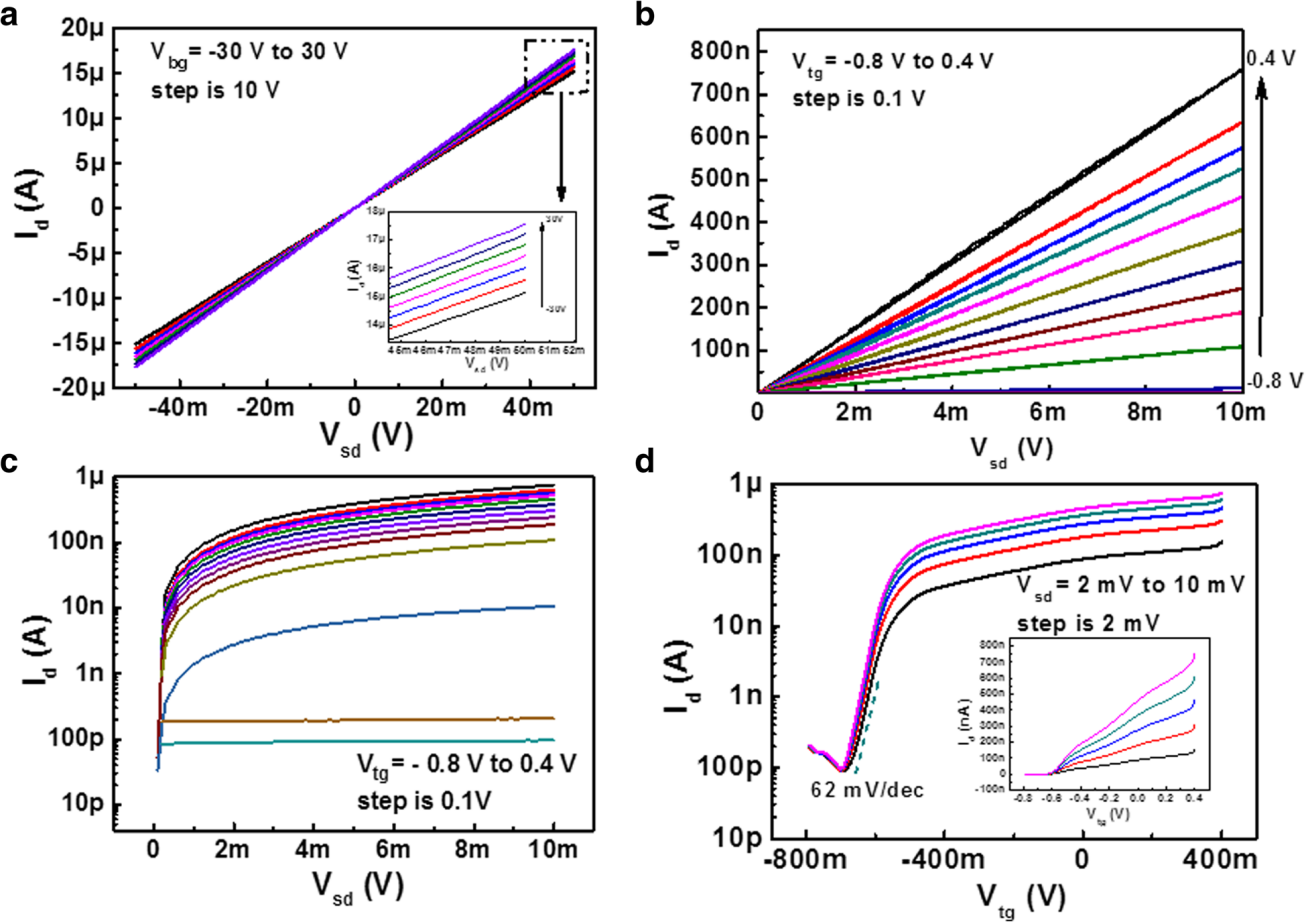
Output and transfer characteristic of SnSe_2_ FET measured in the dark. *I*_d_ versus *V*_sd_ characteristic of SnSe_2_ FET gated at different back gating voltages *V*_*bg*_ (**a**), at different top gating voltages *V*_tg_ in a linear scale (**b**), and at different *V*_tg_ in a semi-log scale (**c**). *I*_d_ versus *V*_tg_ characteristic of SnSe_2_ FET with *V*_sd_ ranging from 2 mV to 10 mV in steps of 2 mV drawn in a semi-log scale, the inset is a linearly-scaled plot of *I*_d_*-V*_tg_ characteristic (**d**)

The output curve of the FET device under different back gate voltages measured in the dark is shown in Fig. 2a. The linear and symmetric relationship of *I*_d_*-V*_ds_ demonstrates an ohmic contact between the Ti/Au electrodes and the SnSe_2_ channel. From Fig. 2a, we found that the modulation effect of the conductivity of SnSe_2_ by back gate voltage is very slight. The ratio of *I*_d_ between gate voltage 30 and − 30 V is only 1.15 at *V*_ds_ of 50 mV. The current *I*_d_ at the back gate voltage of − 30 V is as large as ~ 1.47 μA at *V*_ds_ of 5 mV, which could not be turned off by back gate voltage. Even increasing the large gate voltage up to 100 V still did not bring the channel into its off state as a result of screening of gated potential by the ultrahigh carriers density in the SnSe_2_, which has been reported in previous Pan’s and Su’s work [12, 13]. According to the semiconductor theory, we can make a rough estimation on the depletion width *W* of a metal-insulator-semiconductor (MIS) structure, which is determined by $W={\left(\frac{2{\varepsilon}_r{\varepsilon}_0{\varphi}_s}{e{N}_D}\right)}^{1/2}$, where *φ*_*s*_ is the surface potential, *N*_*D*_ the donor impurity concentration, and *ε*_*0*_ and *ε*_*r*_ vacuum and relative permittivity, respectively. Taking *φ*_*s*_, *ε*_*r*_, *N*_*D*_ of 1 V, 9.97, and 1 × 10^18^/cm^3^ into the equation as a conservative calculation, the depletion width *W* is about 22 nm, which is much smaller than the thickness of our SnSe_2_ flake (34 nm). So, it is easy to understand no depletion of the electrons by the back gate modulation.

In striking contrast, when using DI water as top gate, the *I*_d_*-V*_ds_ curve exhibits an efficient modulation even with a small gate bias, as shown in Fig. 2b. The current ratio between gate voltages of 0.4 V and − 0.8 V is more than 10^3^, which is more clearly seen from Fig. 2c drawn in a semi-log scale. The transfer curves about SnSe_2_ FET with top gate are shown in Fig. 2d, which shows a typical n-type conductive behavior. The voltage scans from the negative direction to the positive direction with a scanning rate of 10 mV/s. Electric double layer (EDL) in ionic liquid or solid electrolyte possesses a high capacitance and can be used to achieve a very efficient charge coupling in 2D and layered materials. However, slow charge transfer processes due to the large ions in size and mass require low-bias scan rates to maintain equilibrium at the gate-channel interface. In contrast, when using DI water as a dielectric layer, both the H^+^ and OH^−^ ions have smaller size and mass and water has a low viscosity. Therefore, DI water gating via the double layer at the water-semiconductor interface supports much higher voltage sweep rates and responds faster than ionic liquids gating or solid electrolyte gating. The inset is a linearly scaled plot of *I*_d_*-V*_tg_ characteristic. Notably, DI water as a top gate greatly enhances transconductance characteristics of the SnSe_2_ FET. As *V*_tg_ varies from − 0.8 to 0.4 V, *I*_*d*_ changes from 9.5 × 10^−11^ to 7.6 × 10^−7^ A with an on/off current ratio of ∼ 10^4^. The subthreshold swing calculated from the transfer characteristic is ∼ 62 mV/decade. These values are good enough for practical, low-voltage operation of layered metal chalcogenide FETs devices. The mobility *μ* can be calculated using the following equation: $\mu =\frac{d{I}_d}{d{V}_g}\cdotp \frac{L}{W{C}_{H2O}{V}_{sd}}$, where *L* and *W* are the channel length and width (*L =* 2 μm, *W* = 5 μm), respectively, and *C*_H2O_ is the DI water-gate capacitance per unit area. Here, the capacitance of *C*_H2O_ was measured to be 348 nF/cm^2^, for which the detailed calculation is attached in the supplementary material (Additional file 1: Figure S1a and b). The obtained electron mobility is 127 cm^2^/Vs, which is quite good compared with other few-layered 2D materials. The substantially improved modulation effect realized by top gate with DI water as a dielectric layer has ever been reported in Huang’s work [27]. They applied DI water gate on the SnS_2_, MoS_2_, and BP flake and achieved a high on/off ratio, ideal subthreshold swing and excellent mobility. They attributed these improvements to perfectly shield the flake from the ambient adsorbates and passivation of the interface states by the high-*k* dielectric (*ε*_*r*(H2O)_ = 80). The passivation and screening effect provided by DI water is similar to that by other conventional high dielectric materials, like HfO_2_ or Al_2_O_3_ [18, 19]. In addition, the effective coupling between the DI water and the SnSe_2_ through the flake edges seems to play an important role in achieving a high on/off ratio even for a thick flake. Compared with SiO_2_ back gating, DI water gating can effectively reduce the electrical field distance (from few 100 nm to less than 1 nm), so the threshold gate voltage also decreased from several tens of volts to less than 1 V. From the inset image of Fig. 2d, the little current jump at about *V*_tg_ = 0.4 V is possibly caused by the electrolysis of DI water due to its narrow electrochemical window, which has been reported in Huang’s work [27].

**Fig. 3:**
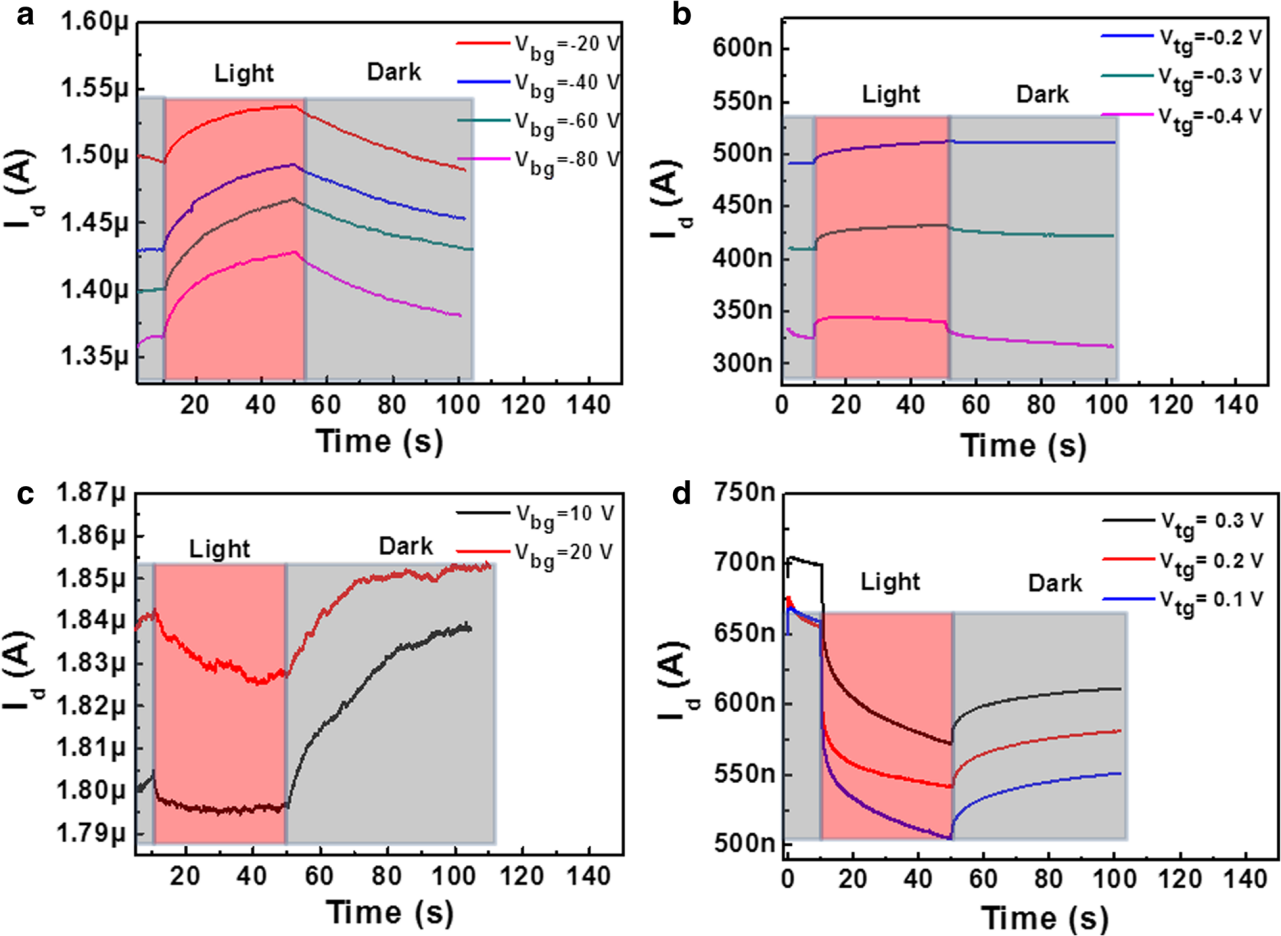
Time dependence of the photoresponse of SnSe_2_ FET biased at *V*_sd_ = 5 mV when applied at different negative back gating voltages *V*_bg_ (**a**), negative top gating voltages *V*_tg_ (**b**), positive back gating voltages *V*_bg_ (**c**), and positive top gating voltages *V*_tg_ (**d**)

The time-dependent photoelectric response of the SnSe_2_ FET controlled by back or top gating is shown in Fig. 3. Interestingly, the SnSe_2_ FET shows a positive photocurrent at a negative gating and a negative photocurrent at a positive gating regardless of gating from back gate via SiO_2_ or from top gate through DI water. From Fig. 3a, we can see the magnitude of photocurrent increases with increasing the negative back gate voltage. When the back gate voltage is − 80 V, the relative photoconductivity (defined as *Δσ/σ*_0_, where *σ*_0_ is the dark conductivity and *Δσ* is the difference between *σ* and *σ*_0_) is 5%. When using DI water as a top gate, we get a similar law as shown in Fig. 3b. With the top gate voltage setting as − 0.4 V, the relative photoconductivity could reach 6%. However, it is easily to see that the response time between the two kinds of gating is quite different. For back gating with SiO_2_ as dielectric, the response time for the rise edge is about 30 s. While for top gating with DI water as dielectric, the response time is only 1.7 s. Here, the 10–90% rise time (or 10–90% fall time) is defined as the response time. The much quicker response speed with DI water gating should be related to the higher carrier mobility (127 cm^2^/Vs) due to the effective screening of impurity or adsorbates scattering. Interestingly, when the gate voltage is positive, the SnSe_2_ film exhibits a negative photoconductivity (NPC) as shown in Fig. 3c and d. It should be emphasized that the gate-dependent bipolar photoconductivity is not induced by the leakage current between the gate and the source. We measured the leakage current of *I*_g_ when applying a positive or negative gate bias, as shown in Additional file 1: Figure S2. The sign of *I*_g_ follows the direction of *V*_gs_ and is just exactly contrary to the sign of drain-to-source photocurrent (*I*_d_). Moreover, the magnitude of *I*_g_ is much smaller than *I*_*d*_, so its impact can be ignored. In NPC of SnSe_2_ FET with H_2_O as dielectric, there are two features which are distinct from positive photoconductivity (PPC). One is the absolute value of the relative photoconductivity gating at positive *V*_tg_ (~ 20%) is eminently greater than that gating at negative *V*_tg_ (6%). The other is the SnSe_2_ FET exhibits a much longer response time (~ 30 s) at positive *V*_tg_ than that at negative *V*_tg_ (1.7 s).

**Fig. 4:**
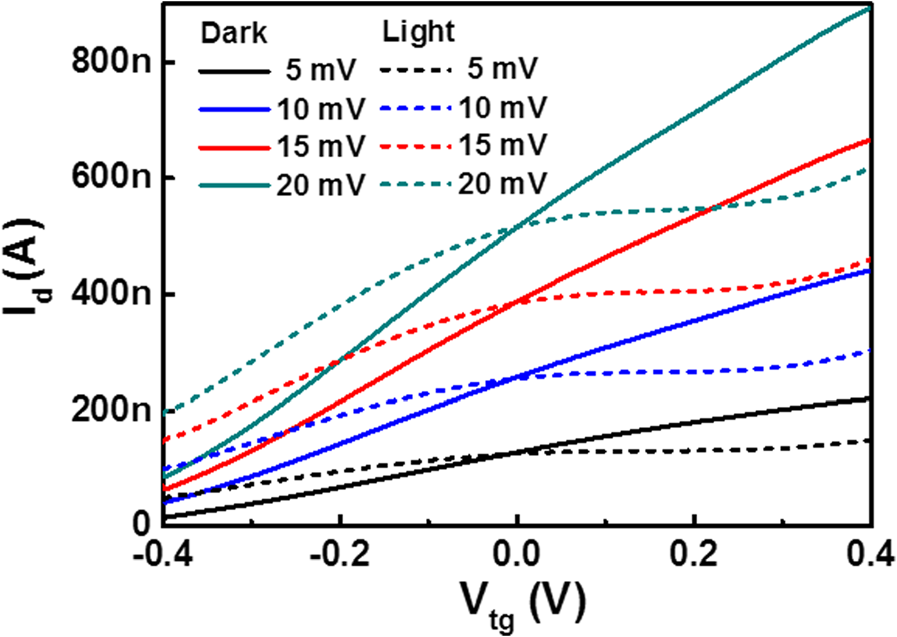
*I*_d_*-V*_tg_ characteristic of SnSe_2_ FET under illumination and in the dark

The negative photoconductivity (NPC) phenomenon has been reported in several semiconductor nanostructures, such as carbon nanotube, InAs nanowire, and ZnSe nanowire [28–30]*.* Oxygen molecular adsorption and photo-desorption are usually suggested to be responsible for NPC effect. However, such an explanation does not apply to our SnSe_2_ system, as oxygen desorption would only lead to higher electron concentration and conductivity. In order to understand NPC effect and the coexistence of NPC and PPC in SnSe_2_, we measured the *I*_d_*-V*_tg_ curves of SnSe_2_ FET under illumination, as shown in Fig. 4. For a clear comparison, the transfer curves in the dark are also added in. We can see the device exhibits a bipolar photoconductivity, which can be switched by gate voltage. The transfer curves measured under illumination and in the dark intersect almost at a gate voltage of 0 V. Therefore, the device shows a positive photoconductivity at a minus gate bias and a negative photoconductivity at a plus gate bias, which is in agreement with the results shown in Fig. 3. As is well known, the conductivity *σ* is determined as *σ* = *neμ*, where *n*, *e*, and *μ* are carrier concentration, electron charge, and mobility, respectively. So, the conductivity is determined by the product of carrier concentration and mobility. In transfer curve under light, the change of transconductance *g*_*m*_ across the zero gate voltage implies an alteration of mobility. From the transfer curves, the mobility of illumination and dark can be calculated as shown in Tables 1 and 2. The mobility of SnSe_2_ in the dark is about 70 cm^2^/Vs, while the mobility under illumination has two values: about 60 cm^2^/Vs at minus gate bias and ~ 4 cm^2^/Vs at plus gate bias. At negative *V*_tg_, the mobility of the light and dark state is almost the same, while the carrier concentration under light excitation is larger than that of dark state. So, the device exhibits a positive photoconductivity. At positive *V*_tg_, the mobility is more than one order smaller than that in the case of negative *V*_tg_, and the decrease in mobility exceeds the increase in carrier concentration and dominates the photoconductivity evolution. Thus, a net negative photoconductivity occurs in replace of the positive photoconductivity.

**Table 1:** The mobility of SnSe_2_ FET with top gating measured in the dark

*V*_tg_ < 0		
*V*_sd_ (V)	*g*_m_ (S)	μ (cm^2^/Vs)
0.005	2.98E−07	68.56
0.01	5.84E−07	67.08
0.015	8.81E−07	67.48
0.02	1.15E−06	65.93

**Table 2:** The mobility of SnSe_2_ FET with top gating measured under illumination

*V*_tg_ < 0			*V*_tg_ > 0		
*V*_sd_ (V)	*g*_m_ (S)	μ (cm^2^/Vs)	*V*_sd_ (V)	*g*_m_ (S)	μ (cm^2^/Vs)
0.005	2.26E−07	52.02	0.005	9.80E−09	2.25
0.01	4.76E−07	54.74	0.01	2.60E−08	2.99
0.015	7.32E−07	56.09	0.015	5.10E−08	3.91
0.02	9.88E−07	56.78	0.02	7.90E−08	4.54

Pai-Chun Wei et al. found NPC effect in a small band gap and degenerate InN film and ascribed it to the depression of the mobility caused by severe scattering from the charged recombination centers [31], which may be applied to our SnSe_2_ system. But why the mobility decreases when the gating bias scans from the negative to the positive voltage is not clear. We believe this phenomenon originates from some in-gap states. The in-gap states can be caused by some point defects, such as Se vacancies. Under illumination, the in-gap states below *E*_*f*_ will trap some photogenerated holes and become positively charged scattering centers. With *V*_tg_ scanning from the negative to the positive bias, more in-gap states dropping below *E*_*f*_ become charged scattering centers, leading to a decline of mobility. Further work is needed to fully understand the mechanism of NPC.

## Conclusions

In summary, SnSe_2_ field-effect transistor (FET) has been fabricated based on SnSe_2_ flake exfoliated from single crystal. With a drop of water as a top dielectric gate, we successfully turned off the device with a high current rejection ratio of ~ 10^4^. Compared with SiO_2_ dielectric gate, the DI water can eminently improve the transport behavior of SnSe_2_ FET with an ideal subthreshold swing of ∼ 62 mV/decade and an excellent electron mobility of ~ 127 cm^2^ V^−1^ s^−1^ at 300 K. Especially, the SnSe_2_ FET exhibits bipolar photoconductivity when the top gate bias scans from − 0.4 to + 0.4 V. The polarity could be switched by the sign of gate voltage. At a negative gate bias, the positive photoconductivity is dominated by the increase in carrier concentration. While at a positive bias, the negative photoconductivity is caused by a sharp drop of mobility. A competition between the carrier concentration and the mobility determines the evolution of photoconductivity. With a facile solution gate method presented in this work, the SnSe_2_ FET demonstrates excellent electric properties and at the same time presents an interesting polarity-switchable photoconductivity, which will open up a new modulate way for high-performance optoelectronic devices.

## Additional file

Additional file 1: Supplementary material for details about calculation of capacitance with DI water as dielectric material and experimental results of leakage current measurements. Figure S1 (a) *I*_d_ versus *V*_tg_ of SnSe_2_ FET biased at different *V*_bg_. (b) *V*_bg_ versus *V*_tg_ derived from *I*_d_ = 240 nA. The red line is a linear fit to the data. Figure S2 Leakage current *I*_g_ of SnSe_2_ FET gated at +*V*_tg_ (a) and at −*V*_tg_ (b). (DOCX 249 kb)

## Abbreviations

2D: Two-dimensional
AFM: Atomic force microscopy
ALMs: Atomically layered materials
DI: De-ionized
FETs: Field-effect transistors
MIS: Metal-insulator-semiconductor
NPC: Negative photoconductivity
PMMA: Polymethyl methacrylate
PPC: Positive photoconductivity
TMDs: Transitional metal dichalcogenides
