# Supplementary material for: SnSe2 Field-Effect Transistor with High On/Off Ratio and Polarity-Switchable Photoconductivity
Source: Nanoscale Res Lett. 2019 Jan 9;14:17. doi: 10.1186/s11671-019-2850-0 (PMC6326916; doi:10.1186/s11671-019-2850-0)
Supplement: Supplementary file 1 — Supplementary material for details about calculation of capacitance with DI water as dielectric material and experimental results of leakage current measurements. Figure S1 (a) Id versus Vtg of SnSe2 FET biased at different Vbg. (b) Vbg versus Vtg derived from Id = 240 nA. The red line is a linear fit to the data. Figure S2 Leakage current Ig of SnSe2 FET gated at +Vtg (a) and at −Vtg (b). (DOCX 249 kb) [file 11671_2019_2850_MOESM1_ESM.docx]

**Supplementary Material**

**SnSe_2_ field-effect transistor with high on-off ratio and polarity-switchable photoconductivity**

Hong Xu,^1,2^ Jie Xing,^1^^[[1]](#footnote-1)^* Yuan Huang^2*^, Chen Ge^2^, Jinghao Lu^1^, Xu Han^1^, Jianyu Du^2^， Huiying Hao^1^, Jingjing Dong^1^, Hao Liu^1^

*^1^School of Sciences, China University of Geosciences, Beijing 100083, China*

*^2^Beijing National Laboratory for Condensed Matter Physics, Institute of Physics, Chinese Academy of Sciences, Beijing 100190, China*

1. **The calculation of capacitance of C_(H2O)_**

The top-gate capacitance was estimated by measuring the change in the transfer characteristic of DI water gated device due to applied different back gate bias *V_bg_* as shown in Fig. S1a. As the back-gate voltage scans from 0 to -1 V, the *I_d_-V_tg_* curve continuously shifts in the positive *V_tg_* direction, while the slope of the *I_d_-V_tg_* curves remains nearly constant in the linear region. We chose *I_d_*=240 nA as a reference and drew a horizontal straight line, which intersects the *I_d_-V_tg_* curves at different *V_tg_*. Then a *V_bg_-V_tg_* curve could be derived as shown in Fig. S1b. Based on the relationship of *C_tg_*·*V*_tg_ = *C_bg_*·*V_bg_* and the capacitance of 100 nm SiO_2_ of 34 nF/cm^2^, the capacitance between SnSe_2_ and DI water is determined to be 348 nF/cm^2^.


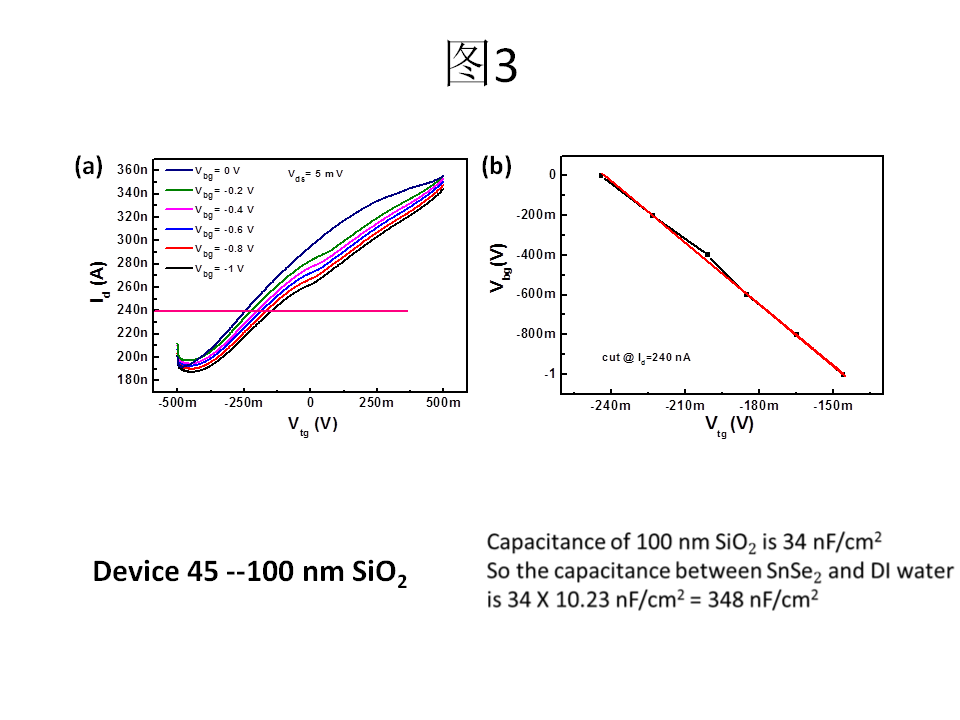


Figure S1 (a) *I_d_* versus *V_tg_* of SnSe_2_ FET biased at different *V_bg_*. (b) *V_bg_* versus *V_tg_* derived from *I_d_*=240 nA. The red line is a linear fit to the data.

1. **The leakage current I_gs_ of SnSe_2_ FET by DI water gating**

Figure S2a and b show the leakage current *I_g_* of SnSe_2_ FET by DI water gating. From Figure S2a, we can see the dark current is less than 300 pA and the photocurrent is only dozens of pA when 0.1 V≤*V_tg_* ≤0.3 V. When at a –*V_tg_* (-0.1 V≤*V_tg_*≤ -0.4 V), the dark current and the photocurrent are both less than 50 pA (as shown in Fig. S2b). These currents are much smaller than *I_d_* when *V_tg_* scans from -0.4 V to 0.3 V.


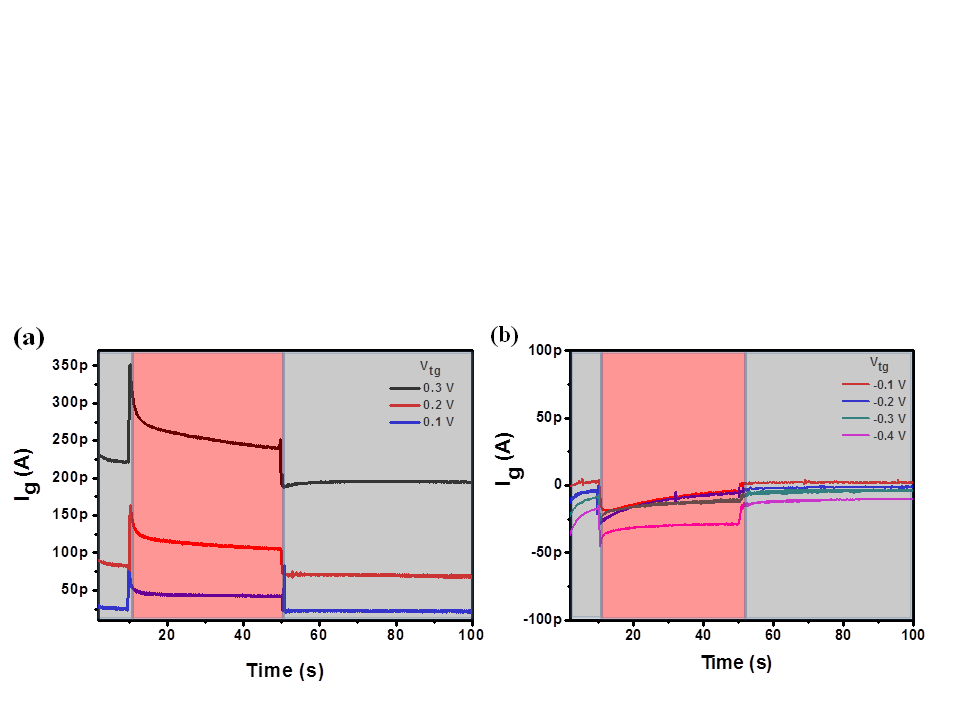


Figure S2 Leakage current *I_g_* of SnSe_2_ FET gated at +*V_tg_* (a) and at -*V_tg_* (b).

1. * corresponding author, Email address: [xingjie@cugb.edu.cn,](mailto:xingjie@cugb.edu.cn,) yhuang01@iphy.ac.cn [↑](#footnote-ref-1)
